# Ex vivo mechanical evaluation of anatomically and mechanically conforming patient-specific lumbar spinal fusion cages designed by full-scale topology optimization

**DOI:** 10.1016/j.xnsj.2026.100890

**Published:** 2026-04-14

**Authors:** Xiaoyu Du, Thijs Smit, Wolfgang Rubin, Charles Cheruparambil, Rahul Shah, Daniel Haschtmann, Benedikt Helgason, Stephen J. Ferguson

**Affiliations:** a Institute for Biomechanics, ETH Zurich, Gloriastrasse 37, 8092 Zurich, Switzerland; b Laboratory of Metal Physics and Technology, ETH Zurich, Vladimir-Prelog-Weg 4, 8093 Zurich, Switzerland; c Interventional Spine Team, Premier Orthopaedic Associates, 352 S. Delsea Drive, Suite C, Vineland, NJ 08360, USA; d Department of Spine Surgery, Schulthess Klinik, Lengghalde 2, 8008 Zurich, Switzerland

**Keywords:** Spinal fusion, Lumbar, Cage, Implant, Ex vivo, Biomechanical testing, Topology optimization, Patient-specific, Human cadaver testing, TLIF

## Abstract

**Background:**

Intervertebral disc degeneration may require spinal fusion surgery using interbody cages. Implant subsidence remains a significant complication, largely attributed to the stiffness mismatch between interbody cages and vertebral endplates. While porous cage designs aim to reduce their stiffness, they do not account for patient-specific anatomical and mechanical variability. This study aims to evaluate whether patient-specific, topology optimized interbody cages improve load distribution and decrease subsidence risk compared with off-the-shelf cages in a human cadaveric mechanical model.

**Methods:**

Sixteen functional spinal units (FSUs) were allocated by bone quality to topology-optimized devices (TOD) or off-the-shelf (OTS) cage groups. Both cage types were designed for a TLIF approach (TOD by a previously developed topology optimization method) with the same footprint and additively manufactured from medical-grade titanium alloy. Endplate contact was assessed using pressure-sensitive film. FSUs underwent axial compression to failure at 1 mm/min with optical displacement tracking. Failure (subsidence) force is defined as the first local maximum of the force–displacement curve.

**Results:**

Pressure film analysis demonstrated broader and more uniform endplate contact for TOD cages, whereas OTS cages exhibited concentrated edge loading. Failure (subsidence) force and integral volumetric bone mineral density (vBMD) were positively correlated for both cage groups. Paired comparisons showed a significantly higher failure (subsidence) force (increase of median force by 97.6%) for TOD implants (median force: 876.5 N) compared to OTS implants (median force: 443.7 N; p = .03).

**Conclusions:**

Patient-specific topology optimized interbody cages improved endplate conformity and load distribution, resulting in higher resistance to subsidence compared to conventional off-the-shelf cages. These findings suggest that patient-specific topology optimized interbody cages may offer a biomechanically advantageous solution for spinal fusion, especially in patients with compromised bone quality, with the potential to reduce implant subsidence and improve clinical outcomes.

## Introduction

Cage subsidence following instrumented lumbar spinal fusion remains a major contributor to treatment failure, particularly in transforaminal lumbar interbody fusion (TLIF) procedures [1]. Interbody cages used in TLIF are typically manufactured from either titanium alloys or polyetheretherketone (PEEK) [2,3]. Titanium implants offer excellent strength, well-established biocompatibility, and favorable surface characteristics for bone on-growth; however, their high stiffness relative to vertebral bone can lead to an increased risk of cage subsidence [4,5]. Conversely, PEEK provides an elastic modulus closer to stiff cancellous bone and is radiolucent for imaging, but it is inherently bioinert and may demonstrate limited osseointegration [6]. Regardless of the material choice, implant subsidence remains a significant complication, with reported rates ranging from 16% to 70% [7,8].

Cage subsidence is a multifactorial phenomenon reflecting mechanical failure of the vertebral endplate due to localized load transfer and stress concentrations at the implant–endplate interface [9,10]. These stresses are largely driven by the mismatch between implant stiffness and vertebral body strength, as well as by the geometrical mismatch, including incongruence between implant geometry and vertebral endplate morphology. Additionally, the relatively small footprint of TLIF cages compared with anterior lumbar interbody fusion (ALIF), limits the load distribution across the endplate and into the vertebra bodies [11,12].

To address this challenge, recent research has shifted toward modifying the implant structural architecture rather than altering bulk material choice, as material selection is constrained by biocompatibility and regulatory requirements. Porous lattice structures, including gyroid, diamond, and other triply periodic minimal surface (TPMS) configurations, have been explored to reduce effective stiffness of the cage and promote bone in-growth. For example, Bozyigit et al. [13] designed a kidney-shaped interbody fusion cage accordingly. The effects of 3 lattice structures, face-centered cubic (FCC), body-centered cubic (BCC) and diamond were studied by finite element analysis. Lower von Mises stresses were observed in the BCC structure. Zhu et al. [14] evaluates the biomechanical efficacy of personalized porous fusion cages, featuring Gyroid (G-Cage) and Voronoi (V-Cage) architectures against classic off-the-shelf (C—Cage) and personalized (P-Cage) designs. The results demonstrated that the V-Cage, with its biomimetic Voronoi porous structure, outperforms other designs by significantly reducing the cage stress and endplate stress when compared to the off-the-shelf C—Cage. However, the designs of lattice structures are limited by their generalized, pattern-based nature and do not account for patient-specific variation in bone quality, local anatomy or the varying load bearing capacity of the vertebra adjacent to the cage.

The authors recently developed a Topology Optimization (TO) strategy to determine the optimal distribution of material within a design domain based on patient-specific TLIF geometry and taking the mechanical response of the adjacent vertebra into account. This leads to an improved load distribution, reduce strain concentrations in the vertebra bodies and endplates with the goal of reducing the risk of cage subsidence [15]. In the authors’ prior work [16], this new TO strategy is applied to design Topology Optimized Devices (TOD) for 7 patients, and tested these in-silico to compare the subsidence risk with off-the-shelf implants (OTS). The study showed an 89% median subsidence risk reduction by using TOD for titanium and a 94% median reduction for PEEK versus OTS designs.

Building upon these findings, the present *ex vivo* biomechanical study aimed to evaluate whether a topology optimized, patient-specific TLIF implant (TOD) provided superior mechanical performance compared to conventional off-the-shelf lattice designs (OTS). The experiments were conducted on human cadaveric spines to assess the potential of TOD implants to mitigate subsidence risk and improve the biomechanical environment for spinal fusion. We hypothesize that TODs show higher failure (subsidence) force in the force-displacement curve resulting from mechanical compression testing compared to OTS devices.

## Materials and methods

### Sample preparation

**Fig. 1:**
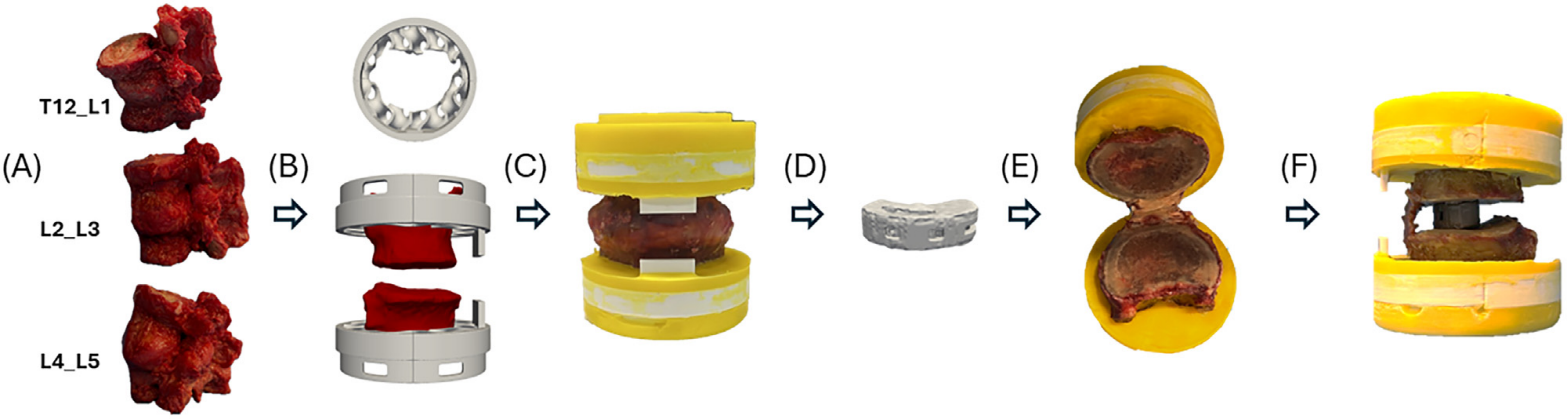
Overview of specimen preparation process: (A) Dissection of T12-L1, L2-L3 and L4-L5 FSUs and removal of spinal process. (B) Custom 3D-printed clamp to ensure alignment. (C) Potting using PMMA. (D) Implant design and manufacture. (E) Endplate preparation, removing cartilaginous endplates and leaving bony endplates intact. (F) Positioning the cage at the designed position.

Six fresh-frozen human cadaveric lumbar spines (3 female, 3 male) were acquired from a registered donor bank (Anatomical Gifts Register, USA) with informed consent provided by next of donor kin. Ethical approval (2025-00307) for this study was granted by the Zurich Cantonal Ethics Committee. Each specimen underwent dual-energy X-ray absorptiometry (DXA) scanning using a clinical system (Horizon Ci; Hologic Inc., Marlborough, MA, USA) to determine vertebral T-scores. From each donor, 3 Functional Spinal Units (FSUs: T12–L1, L2–L3, and L4–L5) were harvested. Specimens were thawed and all surrounding soft tissues were carefully dissected, spinous and transverse process were removed, while the intervertebral discs (IVDs) and a layer of adjacent soft tissue were preserved to maintain anatomical integrity (Fig. 1A).

A summary of the specimen characteristics is provided in Table 1. In 2 donors (4 and 5), only the L2–L3 and L4–L5 FSUs are available, yielding a total of 16 FSUs for inclusion. Based on the T-score, FSUs are evenly allocated into 2 groups: the TOD group and the OTS control group. The mean T-scores were − 1.02 and − 1.26 for the TOD and OTS groups, respectively (p = .85).

**Table 1:** Summary of specimen characteristics as received. T-scores obtained from DXA.

FSU ID	Donor ID	DONOR abbreviation	Segment	Gender	Weight (kg)	Height (m)	Age	T-score upper	T-score lower	WHO class	Implant type
1	1	NC47	L4-L5	F	49.8	1.55	93	−3.5	NA	Osteoporotic	TOD
2	1	NC47	L2-L3	F	49.8	1.55	93	−3.9	−4.2	Osteoporotic	OTS
3	1	NC47	T12-L1	F	49.8	1.55	93	NA	−3.8	Osteoporotic	TOD
4	2	NY24	L4-L5	F	49.8	1.55	84	−0.9	NA	Normal	OTS
5	2	NY24	L2-L3	F	50.3	1.57	84	−1	0.1	Normal	TOD
6	2	NY24	T12-L1	F	50.7	1.60	84	NA	−1.9	Osteopenic	TOD
7	3	VA73	L4-L5	F	65.7	1.63	64	−0.8	NA	Normal	TOD
8	3	VA73	L2-L3	F	65.7	1.63	64	−1.7	−1.4	Osteopenic	OTS
9	3	VA73	T12-L1	F	65.7	1.63	64	NA	−1.9	Osteopenic	OTS
10	4	PA80	L4-L5	M	45.3	1.73	65	−0.7	NA	Normal	OTS
11	4	PA80	L2-L3	M	45.3	1.73	65	−1	−1.4	Osteopenic	TOD
12	4	PA80	T12-L1	M	45.3	1.73	65	NA	−1.2	Osteopenic	OTS
13	5	VA21	L4-L5	M	47.6	1.68	67	5.8	NA	Normal	TOD
14	5	VA21	L2-L3	M	47.6	1.68	67	1.9	2.8	Normal	OTS
15	6	MD73	L4-L5	M	147.2	1.83	62	−2.3	−2	Osteopenic	OTS
16	6	MD73	L2-L3	M	147.2	1.83	62	−2.3	−2.3	Osteopenic	TOD

### Preparation and CT imaging

Each FSU was imaged using a Scanco 100 micro-CT scanner (Scanco Medical AG, Switzerland) at 90 kVp and an isotropic voxel size of 200 µm, providing higher spatial resolution than standard clinical CT to enable detailed assessments related to the current study. Customized 3D-printed clamps were engineered to control the alignment of the vertebra during potting and ensure consistent follower load application during subsequent mechanical testing [17] (Fig. 1B). The clamps were fabricated using a material extrusion process on a 3D printer (Bambu Lab P1S, China) at 90% infill density, employing 1.75 mm polylactic acid (PLA) filament.

Potting was performed using polymethyl methacrylate (PMMA) bone cement (Beracryl, Switzerland), prepared by mixing 65 g of powder with 50 mL of monomer according to the manufacturer’s instructions. The cement was vigorously stirred before pouring, embedding the vertebrae to approximately 50% of their height (Fig. 1C). Curing occurred within approximately 25 minutes. Following potting, each specimen was rescanned using micro-CT (with identical settings as described above) to obtain the images used for implant design.

Throughout the experimental process, the FSUs were stored frozen and were transferred to a refrigerator 24 hours prior to each step (potting, CT imaging, implantation, postimplantation CT imaging, and mechanical testing) to allow controlled thawing.

### Implant design and manufacturing

Postpotting FSU micro-CT scans were segmented (3D Slicer, https://www.slicer.org/), and the vertebrae were extracted. For each FSU, Hounsfield Units (HU) were mapped to volumetric Bone Mineral Density (BMD) using asynchronous calibration with phantoms (hydroxyapatite of know density) scanned before and after each micro-CT scan. The phantom scans were used to establish a linear HU-BMD conversion equation. Subsequently, a resampling (3D Slicer, B-Spline method) of the image to a voxel size of 300 µm was done. The off-the-shelf (OTS) cage geometry and the TOD design domain (anatomically conforming) were based on a commonly used, 9-mm high, curved TLIF cage design including a bone graft hole, and a pivoting inserter interface. For the OTS implant a center-cube lattice infill was included. Standard implant dimensions of 32 mm in length and 10 mm in width were used for both OTS and TOD design domains to ensure an equal footprint. Implant positioning, as well as OTS cage lordosis angle, were determined according to standard TLIF surgical procedures and verified by 2 fellowship-trained spine surgeons. The TOD cages were obtained by topology optimizing the implant design in the anatomically conforming TOD design domain using the previously developed TO strategy [14].

After completion of both the OTS and TOD implant designs, all implants were additively manufactured on a TruPrint 1000 (TRUMPF, Johann-Maus-Str. 2, Ditzingen, Germany) using titanium grade 23 alloy powder with a particle size range of 15–45 µm (Oerlikon Metco AG, Rigackerstrasse 16, 5610, Wohlen, Switzerland) and a layer thickness of 20 µm. The manufacturing process is FDA-approved. Following fabrication, standard postprocessing procedures (support removal, heat treatment, surface finishing) were performed according to the manufacturer’s validated protocol (Amnovis BV, Aarschot, Belgium).

### Pressure film testing

Pressure measurement film (Fujifilm, Japan) LLW was used to qualitatively evaluate contact between the implant and the endplate. TOD and OTS implants were inserted between the endplates at the predefined implant positioning. One side of the assembly was fixed using adhesive applied around the implant, while the pressure film was inserted on the opposite side between the implant and the endplate. A C-clamp was used to apply mild pressure, after which the film was removed and placed in a custom holder for imaging and analysis. Areas of contact were indicated by red coloration on the film, providing a qualitative visualization of implant–endplate contact patterns.

### Mechanical testing

**Fig. 2:**
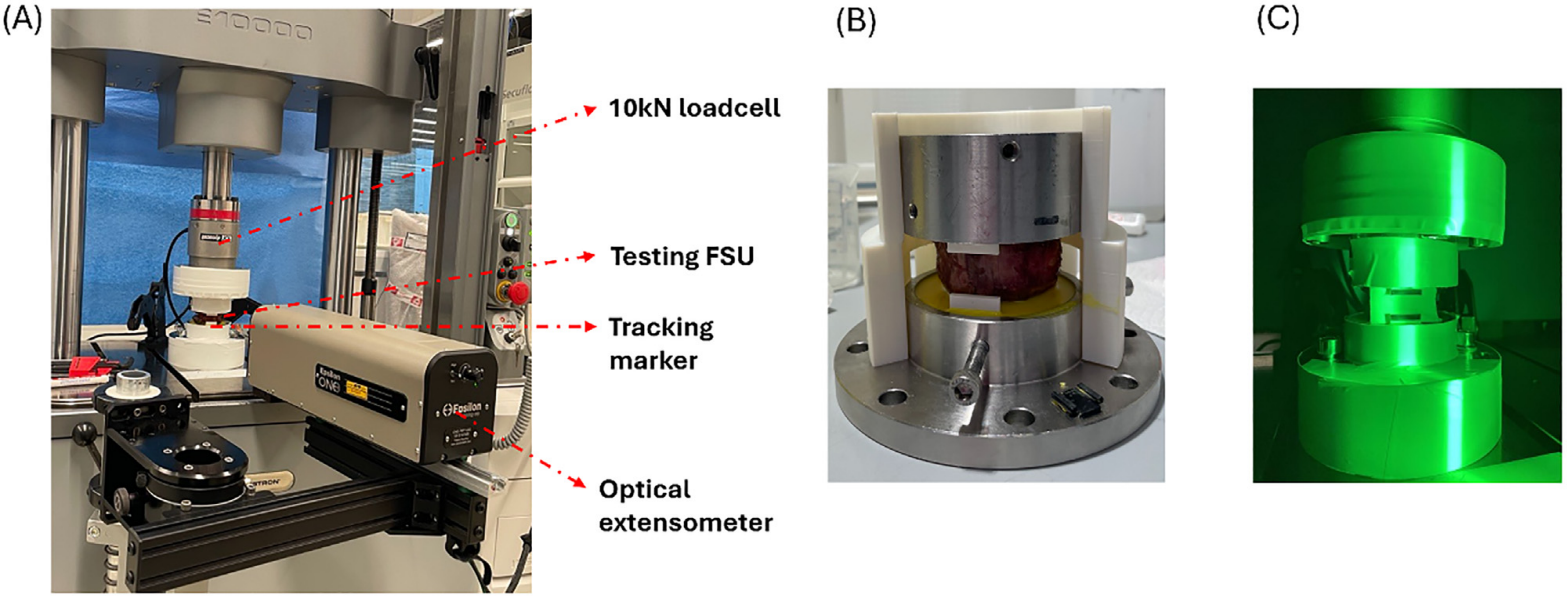
Mechanical test setup: (A) Instron setup with an optical extensometer. (B) Potting sample setup. (C) Tracking lines in the clamp.

To compare the mechanical responses of the FSUs with TOD and OTS implants, compression tests were conducted using an Instron E10000 ElectroPuls system (Instron, UK) (Fig. 2A). Endplate preparation was done according to standard surgical procedures: removing the disc and cartilage endplates but leaving the bony endplates intact. The 3D-printed implants were positioned in the initially designed predetermined locations, and micro-CT scans (using identical settings as described above) were performed to verify and document correct implant positioning prior to mechanical testing. The specimen was placed in a custom cup with an inner diameter of 70 mm and a height of 25 mm, which was attached to the test frame (Fig. 2B). A 10 kN load cell was used, and a preload of approximately 20 N was applied to each sample prior to testing. Compression was applied under displacement control at a rate of 1 mm/min until vertebral fracture, with a final displacement of 6 mm. To avoid errors due to machine compliance, displacement was measured directly on the specimen using an optical extensometer (Epsilon Technology, USA). For this purpose, the custom clamp within the potting fixture was painted black to provide a tracking line for the extensometer (Fig. 2C).

### Post-test micro-CT scan

The FSUs with implants were scanned using micro-CT (with identical settings as described above) to assess subsidence and fracture crack location.

### Data evaluation and statistical analysis

Following mechanical testing, force-displacement curves were generated. Failure (subsidence) force was defined as the first local maximum on the force-displacement curve [18]. Bone quality of each vertebra was assessed from the post potting micro-CT images by calculating the integral volumetric Bone Mineral Density (vBMD), including the endplates and cortical bone [19]. Bone quality was classified according to diagnostic thresholds for integral vBMD: osteoporosis (vBMD < 0.16 g/cm³) and osteopenia (0.16 ≤ vBMD < 0.19 g/cm³) [20]. Failure (subsidence) force was plotted against mean vBMD (mean vBMD of the FSU) for both TOD and OTS implants to evaluate potential correlations. Subsequently, a nonparametric paired Wilcoxon signed-rank test was performed using GraphPad Prism software (version 8.2.0; GraphPad Software Inc., USA), comparing TOD and OTS implants at the L2–L3 and L4–L5 levels. Statistical significance was defined as p ≤ .05 to identify differences between implant types. Spearman’s rank correlation coefficient was used to evaluate the association between failure force and vBMD separately for the TOD and OTS groups, yielding correlation coefficients (*r*, −1 ≤ *r* ≤ 1) and corresponding *p* values indicating the statistical significance of each correlation.

## Results

### Bone quality distribution

The donors exhibited a wide range of ages, from 62 to 93 years (mean age: 76.50 and 70.50 years for the TOD and OTS groups, respectively; p = .42), body mass index (BMI; mean BMI: 23.12 and 22.88 for the TOD and OTS groups, respectively; p = .96), and bone quality. No consistent trend in mean vBMD was identified across donors or spinal levels (mean vBMD: 0.169 and 0.164 g/cm³ for the TOD and OTS groups, respectively; p = .96).

A discrepancy was observed between bone quality classification based on T-scores and that based on mean vBMD. Using T-scores derived from dual-energy X-ray absorptiometry (DXA), 3 FSUs were classified as osteoporotic, whereas 7 FSUs were classified as osteoporotic based on mean vBMD. Donors 2 and 6 demonstrated lower bone quality when assessed using CT-derived measures compared with DXA-based classification. This discrepancy is likely attributable to the 2-dimensional, projection-based nature of DXA, which provides an areal measurement averaged over an area, in contrast to micro-CT, which offers higher spatial resolution and a 3-dimensional, localized assessment of vertebral bone. Given these advantages, in addition to the T-Score based classification, CT-derived mean vBMD was used to classify FSUs into normal, osteopenic, and osteoporotic groups. These classifications are summarized in Table 2.

**Table 2:** Overview of FSUs after preparation and micro-CT: bone quality classification based on vBMD compared with classification based on T-score from DXA.

FSU ID	Donor	Segment	mean v-BMD	Classification (v-BMD)	WHO class (T-Score)	Implant type
1	1	L4-L5	0.13	Osteoporotic	Osteoporotic	TOD
2	1	L2-L3	0.13	Osteoporotic	Osteoporotic	OTS
3	1	T12-L1	0.13	Osteoporotic	Osteoporotic	TOD
4	2	L4-L5	0.14	Osteoporotic	Normal	OTS
5	2	L2-L3	0.19	Osteopenic	Normal	TOD
6	2	T12-L1	0.15	Osteoporotic	Normal	TOD
7	3	L4-L5	0.19	Osteopenic	Osteopenic	TOD
8	3	L2-L3	0.18	Osteopenic	Osteopenic	OTS
9	3	T12-L1	0.18	Osteopenic	Osteopenic	OTS
10	4	L4-L5	0.17	Osteopenic	Osteopenic	OTS
11	4	L2-L3	0.17	Osteopenic	Osteopenic	TOD
12	4	T12-L1	0.16	Osteopenic	Osteopenic	OTS
13	5	L4-L5	0.26	Normal	Normal	TOD
14	5	L2-L3	0.22	Normal	Normal	OTS
15	6	L4-L5	0.14	Osteoporotic	Osteopenic	OTS
16	6	L2-L3	0.13	Osteoporotic	Osteopenic	TOD

### Implant design

**Fig. 3:**
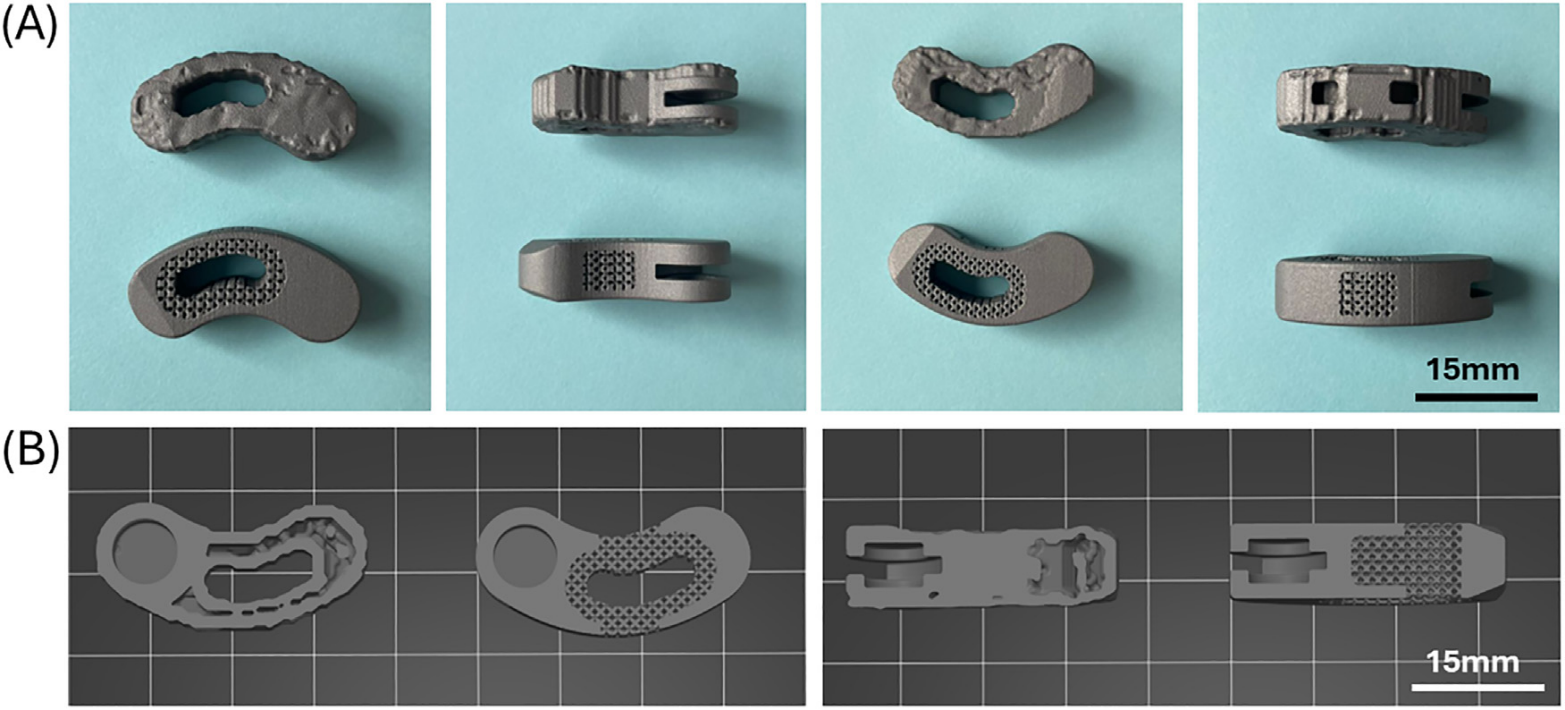
Representative images of the final implant designs. (A) Upper row: TOD implant shown from different sides; lower row: OTS implant shown from different sides. (B) Cross-sectional images of the TOD and OTS implants.

Fig. 3 illustrates a selection of the final implant designs. As in the prior published designs, the TOD designs resulted in a box-like structure, conforming to the endplates, with patient-specific internal struts [14]. These struts appeared in the design to guide load transfer through the implant, influence the local stiffness of the implant, and provide internal structural reinforcement. Cages from both groups had similar heights and identical footprint areas.

### Implant placement and contact with endplates

**Fig. 4:**
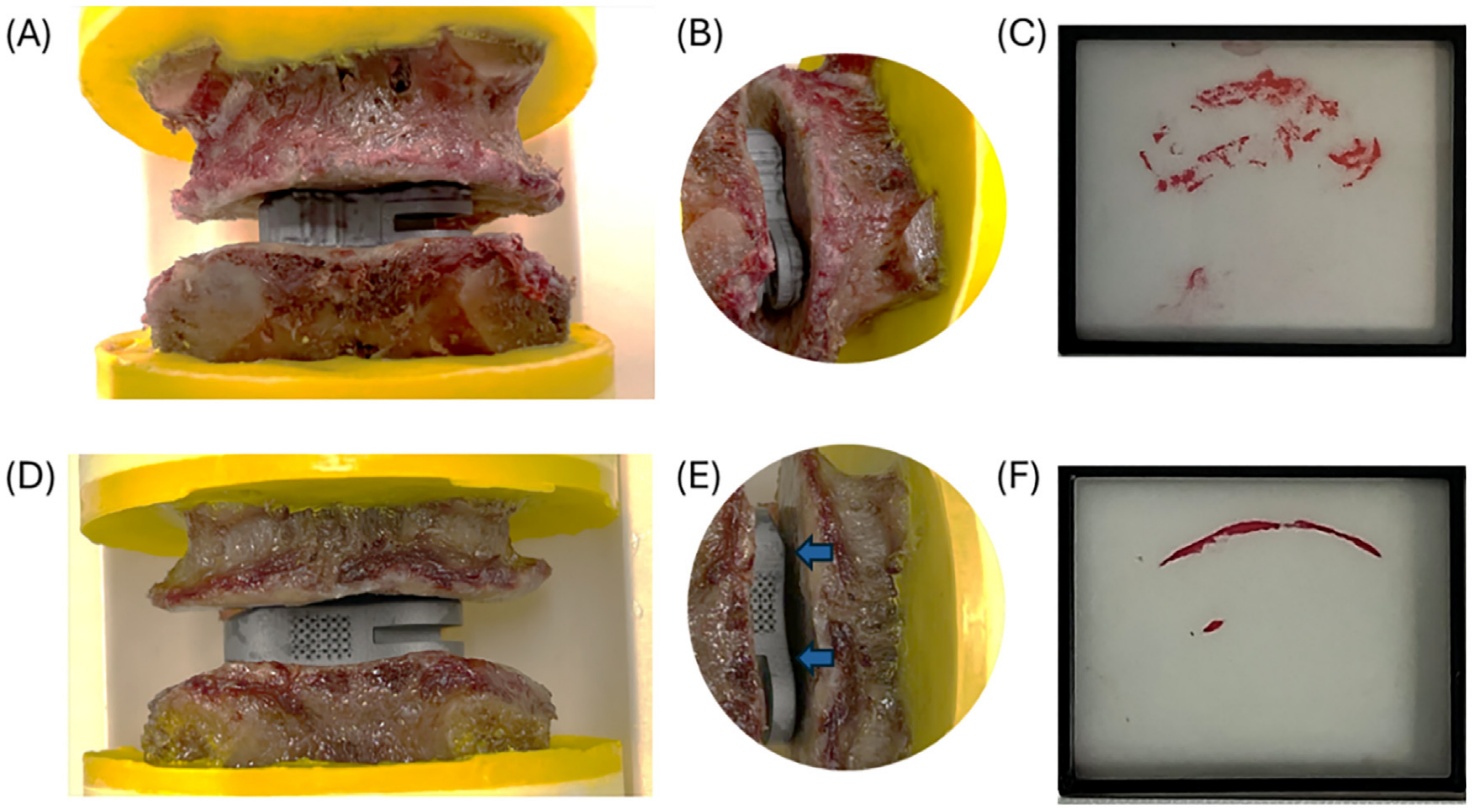
Pressure film test of the implant within FSUs. (A, B) Representative TOD implant with FSU. (C) Pressure film results for the TOD implant. (D, E) Representative OTS implant with FSU; blue arrows indicate gaps between the vertebral endplates and the implant. (F) Pressure film results for the OTS implant.

TOD and OTS implants were positioned between adjacent vertebrae according to the planned implantation location. TOD implants demonstrated close conformity to the endplate morphology, whereas OTS implants exhibited gaps between the implant and endplate. This mismatch was more pronounced in specimens with curved or nonplanar endplates (Fig. 4). Pressure sensitive film placed between the implant and vertebral endplates revealed broader and more uniformly distributed contact for TOD implants, indicated by homogeneous color patterns (Fig. 4C). In contrast, OTS implants showed localized regions of higher pressure, predominantly at the implant edges, indicating concentrated edge loading and reduced contact area (Fig. 4F).

### Mechanical testing results

**Fig. 5:**
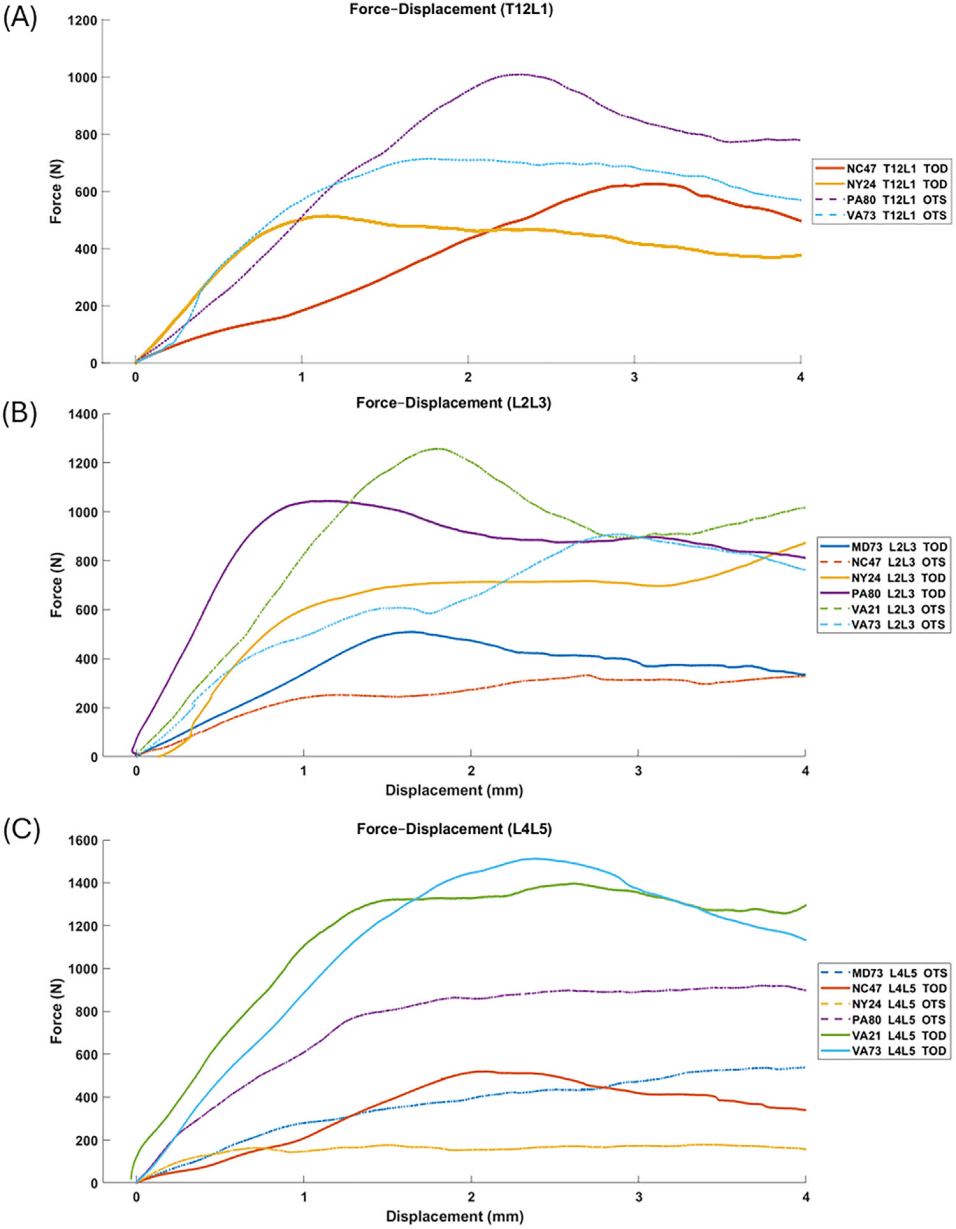
Force–displacement curves for all specimens across the different functional spinal unit (FSU) levels: (A) T12–L1, (B) L2–L3, and (C) L4–L5. Displacement was measured using an optical extensometer. Specimens from the same donor are represented by the same color; solid lines correspond to TOD implants, whereas dashed lines represent OTS implants.

**Fig. 6:**
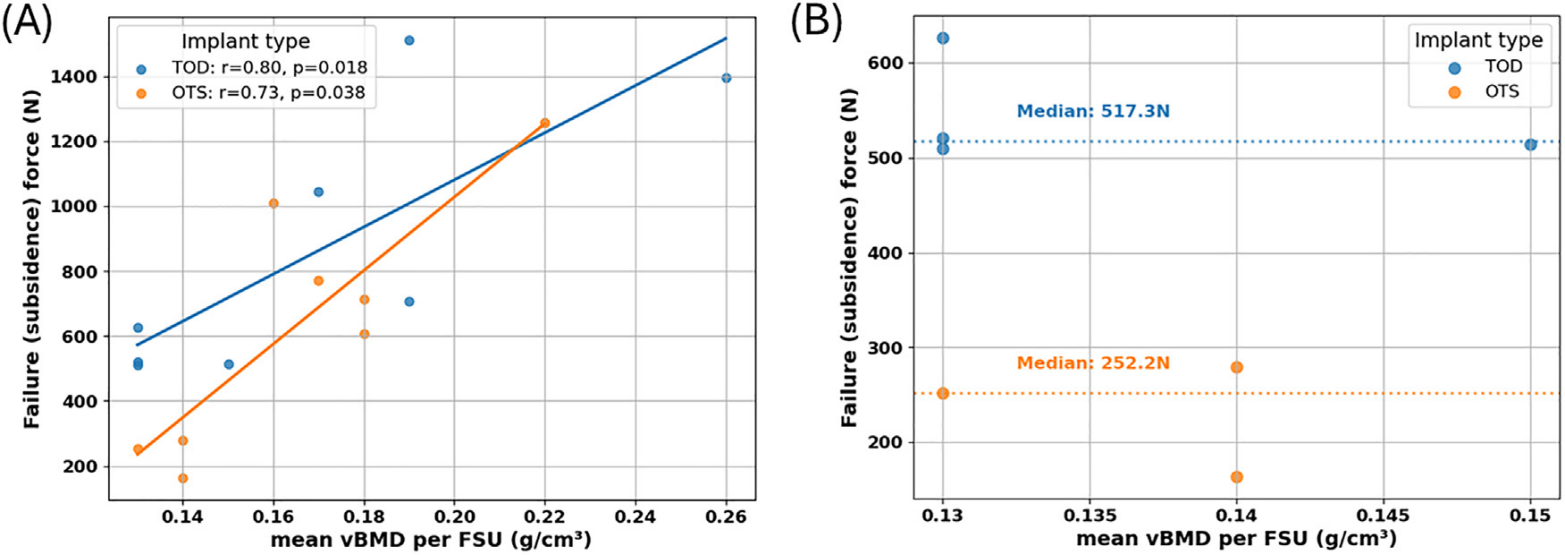
Mechanical testing results summarized: (A) Failure (subsidence force) versus mean vBMD per FSU for all samples. (B) Failure (subsidence force) versus mean vBMD for the osteoporotic group.

Force–displacement curves for all specimens across the different FSU levels are presented in Fig. 5. Each donor is represented by a unique color, with solid lines corresponding to TOD implants and dashed lines to OTS implants. All curves demonstrate an initial linear elastic response, followed by yielding and eventual failure. When comparing the force-displacement curves at each level, no consistent trend was observed, likely reflecting the interdonor variability in bone quality. As shown in Fig. 6A, for both implant designs, failure (subsidence) force correlated with vBMD (r = 0.80, p = .02 for the TOD group and r = 0.73, p = .04 for the OTS group). In osteoporotic FSUs, TOD cages demonstrated approximately a twofold increase of resistance to subsidence compared to OTS cages, with a median failure (subsidence) force increase of 105.1% (median: 252.2 N for OTS vs. 517.3 N for TOD; Fig. 6B).

**Fig. 7:**
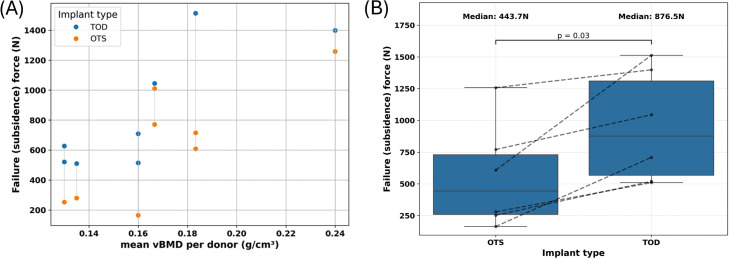
Mechanical testing results summarized: (A) Failure (subsidence force) versus mean vBMD per donor. Averaging the mean vBMD for all FSUs per donor allows for an easier comparison per donor. (B) Paired comparison for each donor between the OTS and TOD implants at the L2–L3 and L4–L5 levels.

For each donor, FSUs implanted with TOD cages exhibited higher failure forces than those implanted with OTS cages (Fig. 7A). Paired comparisons within each donor showed a significant increase in the median failure (subsidence) force of 97.57% for TOD implants (median: 876.5 N) compared with OTS implants (median: 443.7 N; p = .03) (Fig. 7B).

### Special case: FSU with vertebral fracture

**Fig. 8:**
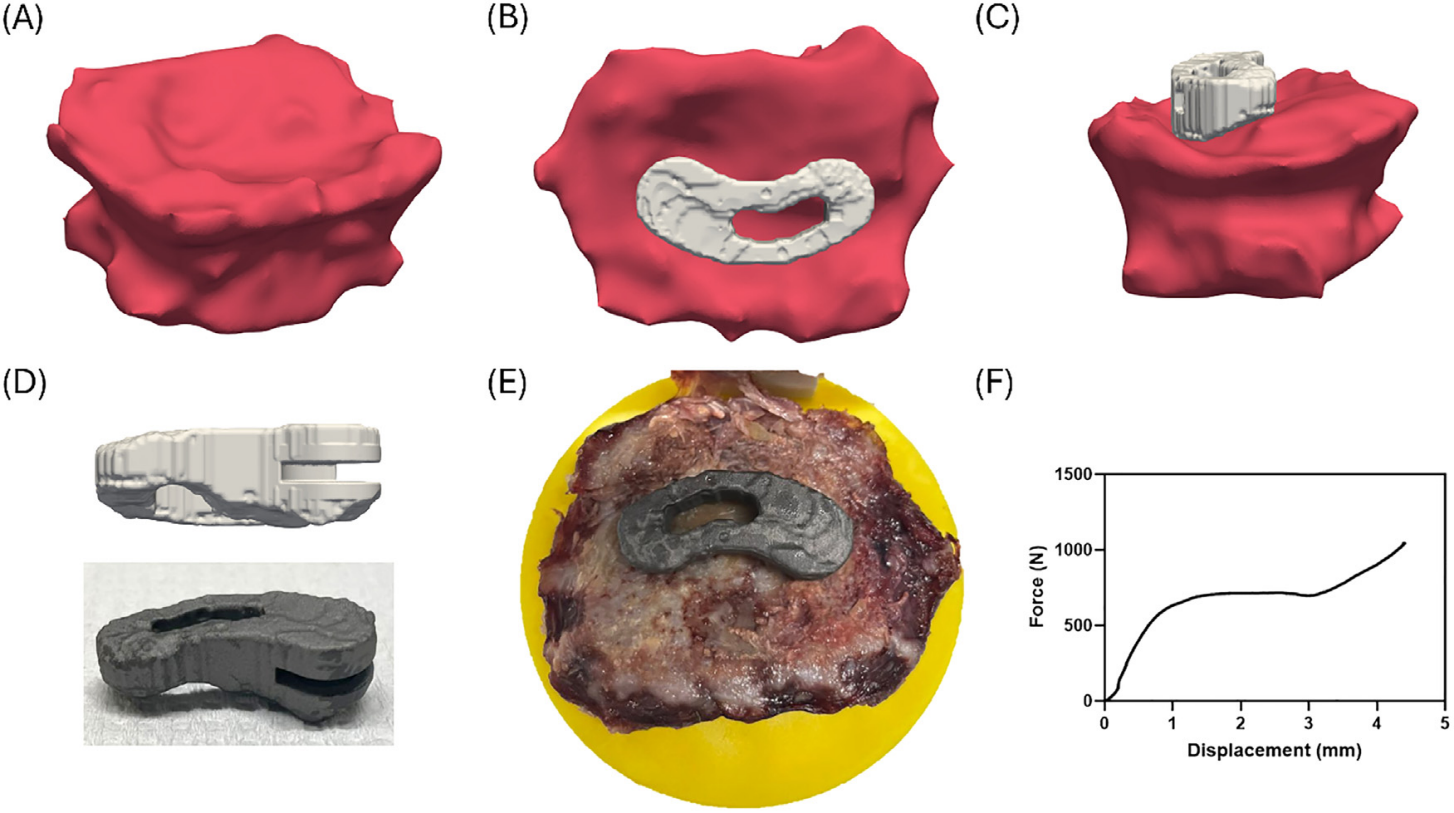
Special case with a considerably degenerated FSU (Donor 2, L2-L3). (A–C) TOD implant positioned on the vertebrae from the model. (D) TOD implant design and 3D-printed titanium implant. (E) Manufactured implant placed on the vertebrae according to pre-operative planning. (F) Force–displacement curve of mechanical testing.

One FSU exhibited considerable endplate degeneration (FSU ID 5). In this case, a TOD implant was used, as its patient-specific geometry allowed it to conform to the remaining vertebral anatomy (Fig. 8C), whereas an OTS implant would not have been able to sufficiently restore the FSU. Mechanical testing demonstrated that, despite the compromised bone structure, the force-displacement curve exhibited behavior similar to the other force–displacement curves related to OTS and TOD (Fig. 8F).

### Failure analysis

**Fig. 9:**
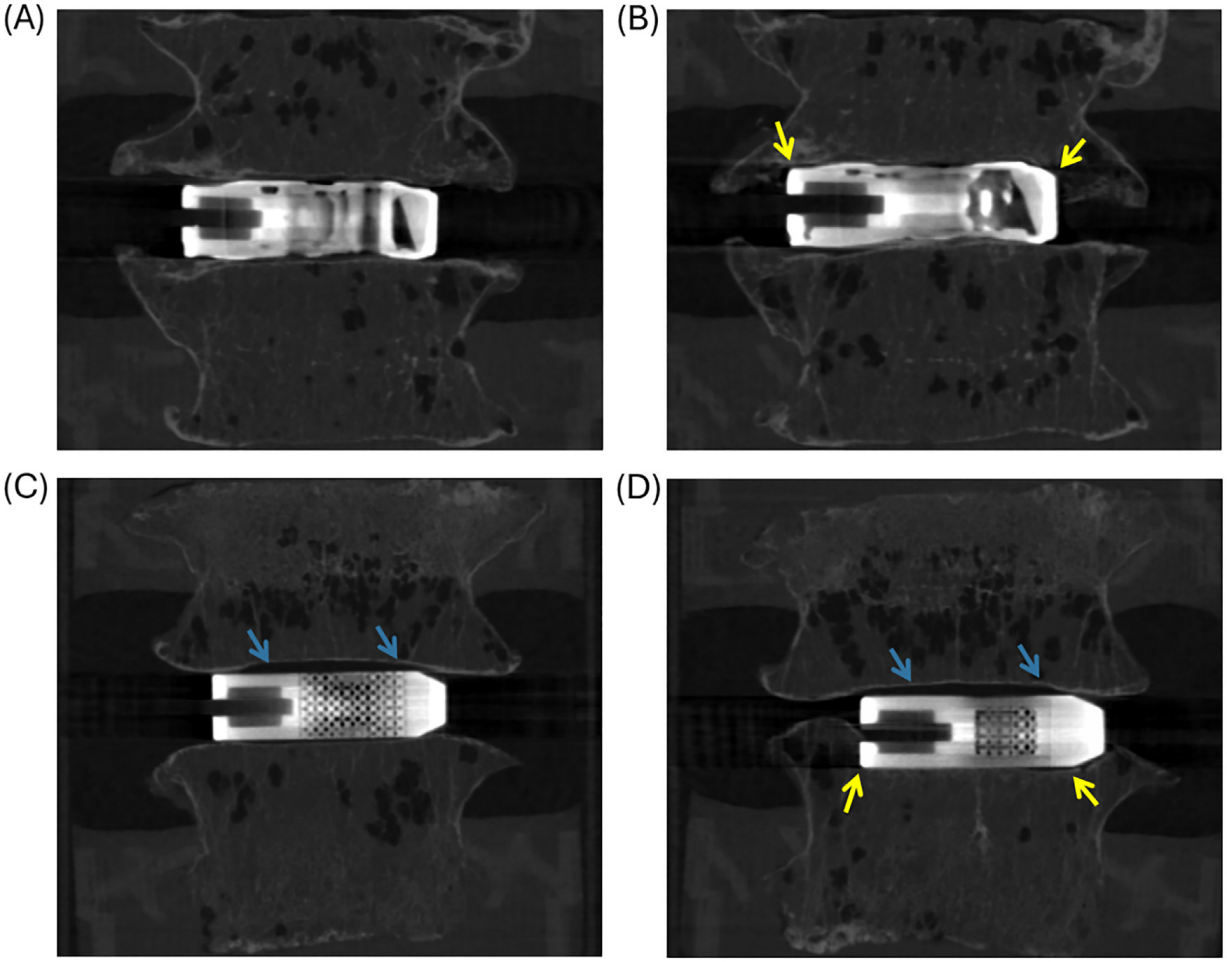
(A, B) Micro-CT images of a representative TOD implant with FSU (Donor 2, T12-L1) before and after testing. In this FSU both upper and lower vertebra had a vBMD of 0.15 g/cm3. (C, D) Micro-CT images of a representative OTS implant with FSU (Donor 2, L4-L5) before and after testing. Yellow arrows indicate the subsidence location, and blue arrows indicate gaps between the OTS implant and the vertebral endplate. In this FSU the upper vertebra had a vBMD of 0.17 g/cm3 and the lower vertebra of 0.10 g/cm3 causing the subsidence to occur in the lower vertebra.

After mechanical testing, all FSUs were rescanned using micro–CT to identify the location and pattern of subsidence. No consistent trend is observed between TOD and OTS implants regarding the location of subsidence. However, in specimens implanted with OTS cages, a persistent gap between the implant and the endplate was observed in some cases, even after 6 mm of compression (Fig. 9).

## Discussion

The aim of this study was to evaluate whether patient-specific, topology optimized interbody cages (TOD) improve resistance to subsidence compared with conventional off-the-shelf (OTS) cages under axial compression. Using human cadaveric functional spinal units, we demonstrated that TOD implants significantly increased failure (subsidence) relative to OTS designs, with a statistically significant median increase of 97.6%. Bone mineral density is a well-recognized determinant of interbody cage subsidence in clinical practice, with patients exhibiting low bone quality demonstrating an increased risk of implant settling and loss of disc height [11,21]. The present findings are consistent with this clinical experience, as the relative advantage of topology-optimized, patient-specific cages was most pronounced in specimens with low mean vBMD.

For mechanical testing, cages were positioned anteriorly to the midline within the intervertebral space. This standardized anterior placement was chosen to ensure symmetric load transfer under pure axial compression and to minimize variability related to unilateral cage positioning. The positioning of the cages was verified by 2 fellowship-trained spine surgeons. As the objective of this study was to compare intrinsic mechanical performance and subsidence resistance between TOD and OTS designs, anterior placement provided a controlled and reproducible testing configuration.

The observed improvement in subsidence resistance can be attributed to 2 primary biomechanical advantages of the TOD design. First, the TOD implant is adapted to the individual endplate morphology with optimized implant–endplate contact area and pressure distribution. The geometric conformity promotes more uniform load distribution, and reduces stress and strain concentrations at implant edges, which are known contributors to endplate fracture and subsidence [22]. This is supported by pressuresensitive film analysis and post-test imaging in our study, which demonstrated broader and more homogeneous endplate contact for TOD implants compared with the localized edge loading observed with OTS designs (Fig. 4). It should be noted that the pressuresensitive film was used for qualitative comparison only, as precise quantitative pressure measurements are difficult to obtain at the implant–endplate interface. The vertebral endplate is inherently nonplanar, and the pressure film lacks sufficient compliance to fully conform to local surface irregularities, which can lead to incomplete contact and inaccuracies in absolute pressure values. Accordingly, the pressure film was used to provide a qualitative visualization of contact patterns and relative pressure distribution trends rather than exact quantitative measurements.

Consistent with these findings, Patel et al. [23] reported that nonconforming implants generate high stress concentrations at the edges of the endplate–implant interface. Similarly, Fernandes et al. [22] demonstrated that improved utilization of the cage contact area enhances load sharing across the endplate and significantly reduces contact stress. These studies support the biomechanical advantages observed with the patient-specific TOD implant in the present study. Moreover, our biomechanical observations are consistent with clinical experience in TLIF procedure, which is often associated with postoperative loss of disc height and segmental kyphosis when nonconforming cages concentrate loads at the endplate margins [24]. The present results suggest that improved implant–endplate conformity may mitigate these mechanisms by promoting more uniform load transfer across the vertebral endplate.

Second, through topology optimization, the internal structure of the TOD implant was designed to achieve spatially varying stiffness and load-bearing capacity [25,26], enabling improved load distribution and mechanical conformity with the adjacent vertebrae. This is particularly important in the presence of compromised bone quality, where excessive local (bone) stresses in the bone–implant structure can accelerate subsidence [27,28]. In a previous work by Lin et al. [29] a structural and mechanical evaluation of a topology optimized titanium interbody fusion cage fabricated using selective laser melting was performed and demonstrated sufficient compressive strength without excessive stiffness, supporting segmental spinal integrity. However, those tests were conducted on the implant alone and did not account for interactions with the surrounding vertebral bone.

In contrast, the present study evaluated implant performance within human cadaveric FSUs of varying BMD. Mechanical testing demonstrated that TOD implants consistently exhibited higher failure (subsidence) forces than OTS implants across a clinically relevant range of bone mineral densities (Fig. 7). Notably, this advantage was most pronounced in specimens with low mean vBMD, representative of osteoporotic bone, where TOD implants showed substantially greater resistance to subsidence (Fig. 6B). These findings indicate that topology optimization can meaningfully reduce subsidence risk by mitigating stress and strain concentrations and improving load transfer, particularly in vertebrae with reduced bone quality.

A limitation of this study was that all experiments were conducted using human cadaveric specimens, which do not capture the effects of biological remodeling, fusion progression, or long-term bone–implant interactions [30]. In addition, the tested functional spinal units represented different spinal levels, which introduces anatomical and biomechanical heterogeneity. Clinical experience indicates that TLIF procedures are most commonly performed at the L4–L5 and L5–S1 levels, where loading demands and failure risk are typically higher than at more cranial levels such as L2–L3 [31]. Consequently, differences in segmental anatomy and the local loading environment may influence subsidence behavior, thereby limiting direct extrapolation of the findings to specific clinical scenarios.

The application of a follower load in cadaveric spinal testing is an established method for simulating physiologic compressive loading along the spinal curvature and approximating *in vivo* biomechanical conditions [17]. Although customized 3D-printed clamps were used to standardize vertebral alignment during potting and to ensure consistent follower load application, intrinsic anatomical differences between motion segments (T12–L1, L2–3, and L4–5) may still affect local load distribution. However, because alignment was mechanically controlled and each specimen served as its own control in the comparison between TOD and OTS cages, the relative differences in subsidence resistance remain valid.

Additionally, loading conditions were limited to axial compression and did not capture complex, multidirectional physiological loads. Nevertheless, axial compression is the primary loading mode experienced by lumbar interbody cages and is therefore an appropriate and widely accepted testing condition. Compression testing is commonly used to characterize the mechanical performance of lattice-based spinal cages, as it enables the evaluation of key mechanical properties such as stiffness, failure (subsidence) force, and ultimate compressive strength, which are essential for assessing implant stability and load-bearing capacity [32,33]. Future studies should incorporate larger and more diverse cohorts, evaluate cyclic and multiaxial loading conditions, and assess long-term biological integration and clinical outcomes associated with anatomically and mechanically conforming, topology optimized interbody implants.

Beyond these limitations, from a clinical perspective, these findings indicate that patient-specific topology optimized interbody cages may offer meaningful advantages for populations at increased risk of implant-related complications, such as elderly or osteoporotic patients. By improving implant–endplate conformity and promoting more uniform load transmission, a TOD has the potential to reduce subsidence, preserve segmental alignment, enhance spinal fusion, and consequently improve clinical outcome while decreasing the likelihood of revision surgery. The observed biomechanical improvements support further translational investigation of patient-specific topology optimized cage designs as a strategy to enhance bone-implant stability and fusion success in high-risk patients.

## Conclusion

In summary, we found that patient-specific topology optimized spinal fusion cages demonstrated improved endplate contact and approximately twofold higher resistance to subsidence compared to conventional off-the-shelf designs. The enhanced performance of the topology optimized design was particularly evident in specimens with low volumetric bone mineral density, highlighting its potential advantage for osteoporotic patients. These findings suggest that optimizing implant topology, rather than altering implant material, may be an effective strategy to reduce subsidence risk in lumbar spinal fusion. Further studies incorporating larger cohorts, complex loading conditions, and clinical outcome measures are warranted to establish the translational relevance of these findings.

## Declaration of generative AI and AI-assisted technologies in the writing process

During the preparation of this work the author(s) used ChatGPT in order to improve readability. After using this tool/service, the author(s) reviewed and edited the content as needed and take(s) full responsibility for the content of the publication.

## Data availability

Data available upon reasonable request.

## Declarations of competing interests

One or more authors declare potential competing financial interests or personal relationships as specified on required ICMJE-NASSJ Disclosure Forms.
